# Insight into the biogenesis of tax nuclear speckles using comparative interactome analysis

**DOI:** 10.1186/1742-4690-12-S1-O35

**Published:** 2015-08-28

**Authors:** Hampus Engstrom, David J Archer, Sucharita Dutta, John O Semmes

**Affiliations:** 1 The Leroy T Canoles Jr. Cancer Research Center, Eastern Virginia Medical School, Norfolk, Virginia, USA

## 

HTLV-1 Tax protein displays pleotropic activities that regulate many aspects of virus biology, host-pathogen interaction and disease development. Tax performs these various functions via protein-protein interactions and sub cellular compartmentalization appears to regulate which activities are engaged. We previously identified a novel nuclear body, Tax Speckled Structures (TSS), the biogenesis of which supports the sequestration of cellular DNA Damage Response factors by Tax to chromatin. To identify cellular proteins that drive localization of Tax to chromatin “speckles”, we conducted a Comparative Interactome Analysis of Tax and a Tax mutant missing the TSS localization signal (Tax-TSLS). Tandem affinity tagged Tax and mutant proteins were expressed in eukaryotic cells. Cytoplasmic, nuclear and chromatin fractions were generated and protein interactomes isolated by affinity purification. Those proteins that preferentially bound full-length Tax over Tax-TSLS were defined as the TSLS-specific interactome. Quantitative Ultra-high resolution tandem mass spectrometry was employed to establish the most comprehensive map of Tax-interacting proteins to date. We employed bioinformatics approaches to compare Tax interactomes and identify the functional pathways and interaction networks unique to proteins that bound the TSLS-defined region. The top functional networks for proteins that bound to the TSLS were involved in chromosomal alignment, congression, segregation, remodeling and modification. The overwhelming majority were known components of four chromatin functional super-complexes condensin I, kinesin, SWI/SNF and NURF. We also examined the protein interactions that were shared by full length and mutant Tax. These included significant interaction with DNA damage, cell cycle check point, NFkB signaling, and protein sorting pathways. A comparative analysis between the sub cellular fractions revealed that DNA damage and checkpoint signaling interactions were enriched in the nucleus/chromatin. These results demonstrate that Tax initiates TSS through binding of chromatin complexes and bridges the recruitment of DNA damage response and checkpoint signaling proteins.

